# "Tips and tricks" in secondary bladder neck sclerosis’ bipolar plasma vaporization approach

**Published:** 2013-09-25

**Authors:** C Moldoveanu, B Geavlete, F Stănescu, M Jecu, L Adou, C Bulai, C Ene, P Geavlete

**Affiliations:** "Saint John" Emergency Clinical Hospital, Department of Urology

**Keywords:** bladder neck sclerosis, bipolar plasma vaporization, transurethral incision/ resection, sclerotic tissue ablation

## Abstract

**Introduction: **Secondary bladder neck sclerosis (BNS) represents a common late complication of prostate surgery, however so far insufficiently assessed in the available literature. More over, the previously attempted and analyzed therapeutic modalities failed to achieve acknowledgement as standard treatment for this particular pathology.

**Methods:** The bipolar plasma vaporization (BPV) was introduced as a viable mean of removing the obstructing scar formation in a gradual fashion. Several "tips and tricks" were described as particularly useful in optimizing the plasma corona vaporization effect. The proper BPV technique is simple and safe while closely relying on certain surgical steps, the simultaneous vaporization and coagulation processes and a superior endoscopic vision. Recent technological advances created the premises for further improvements.

**Results:** The plasma-button vaporization is characterized by a satisfactory surgical speed, remote intraoperative bleeding risks, high-quality endoscopic visibility as well as the achievement of a complete sclerotic tissue removal. Within a short learning curve, a superior final aspect of the prostatic fossa and bladder neck is obtained at the end of surgery. The continuous plasma vaporization mode provides additional technical advantages. The previously described drawbacks of transurethral incision or resection seem to have been overcome by the practical features of the plasma vaporization process.

**Conclusions: **The BPV technique outlines a promising modality of efficiently ablating the obstructing fibrous tissue in secondary BNS patients. The simplicity and safety of the bipolar vaporization approach, together with the thorough obstructing scar removal in a radical fashion, create the premises for a favorable long term BPV clinical outcome.

## Introduction

The present analysis is aimed to address a well-known but yet poorly evaluated late complication of prostate surgery, the secondary bladder neck sclerosis (BNS). While affecting the results of a wide range of interventions (benign prostatic hyperplasia (BPH) endoscopic and open approaches, radical prostatectomy), this adverse event was so far remotely evaluated in the literature [**1**]. 

 On the other hand, despite the various treatment alternatives presented over the years (monopolar [**2**] or bipolar [**3**] encircling resection and bladder neck incision by either cold-knife [**4**] or laser [**5**]), the available literature suffered from the relatively reduced number of cases [**6**], disappointing long tern results in general [**7**] and elevated recurrence rates in particular [**8**]. 

 Based on these premises, the present paper discusses a relatively new technique in bladder neck contracture, the bipolar plasma vaporization (BPV). After already acquiring a substantial degree of therapeutic success in cases of BPH related bladder outlet obstruction [**9**] and large bladder tumors [**10**], the method in question began to be successfully applied in our experience for this particular pathology as well [**11**]. While the short [**12**] and medium [**13**] term results of this type of approach were shown to display a generally favorable clinical outcome, the practical advantages of the procedure as well as the technical means of obtaining them were thought to require further clarification and constituted the object of this paper. 

**Bipolar plasma vaporization – Basic surgical steps**

**Fig. 1:**
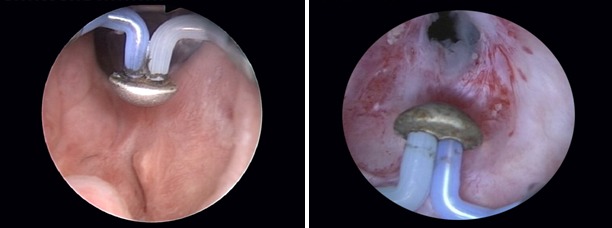
Initial BNS aspect of the prostatic fossa and bladder neck area

**Fig. 2:**
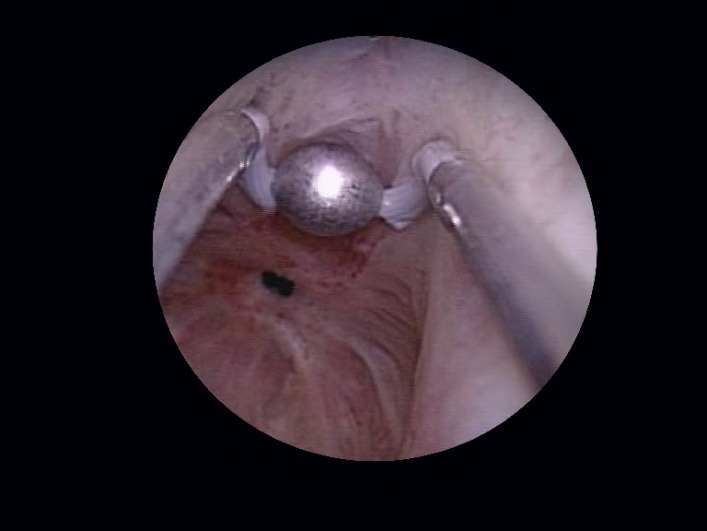
Severely narrowed bladder neck

 At the very beginning of the BPV procedure, a careful endoscopic evaluation of the prostatic fossa and bladder neck area is carried out (**Fig. 1**). In case of a tight bladder neck stenosis only leaving an extremely narrow communication with the bladder, a safety guidewire is inserted through the respective orifice in order to ensure a correct bladder neck opening and to prevent false passages and perforations (**Fig. 2**). 

**Fig. 3:**
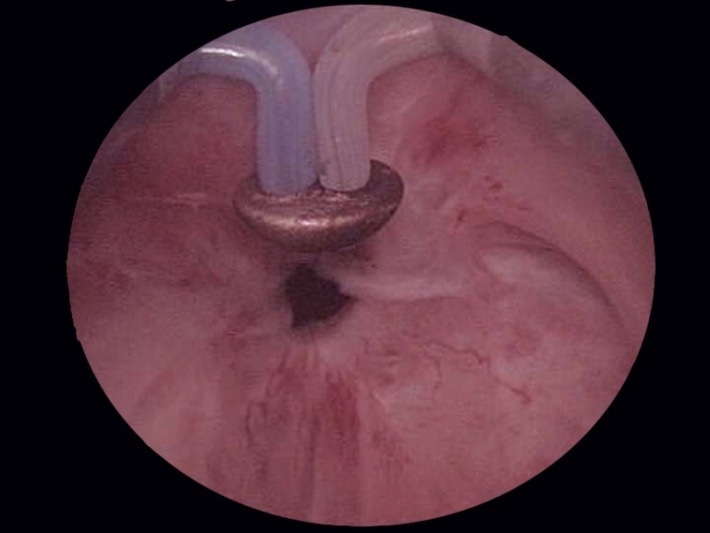
Obstructive diaphragm of sclerotic tissue around the bladder neck circumference

**Fig. 4:**
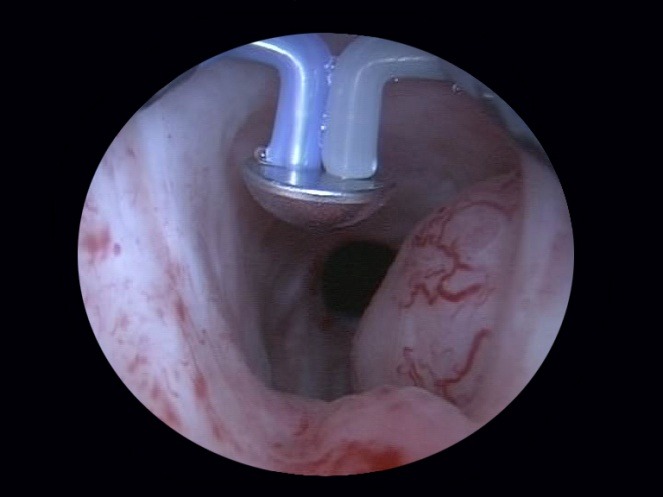
Residual BPH tissue partially occupying the prostatic fossa

**Fig. 5:**
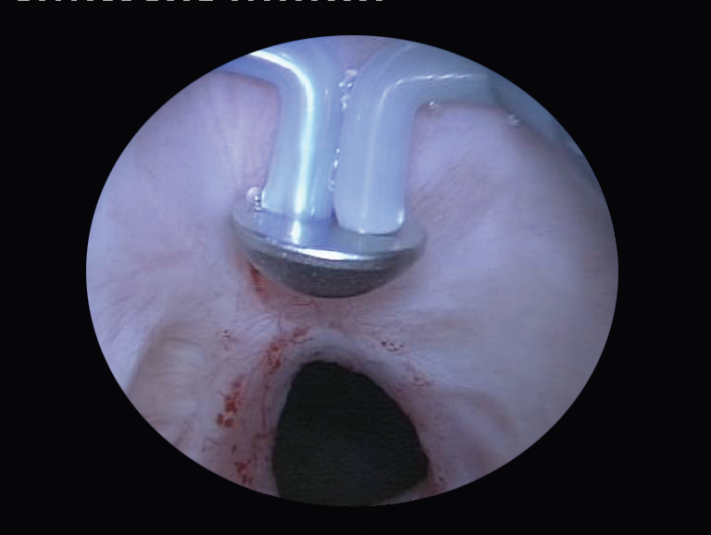
Fibrous formation secondary to standard TUR

While intending to determine the proper vastness of the required vaporization process, this initial assessment analyzes the size and characteristics of the obstructive diaphragm of sclerotic tissue around the bladder neck circumference (**Fig. 3**) as well as the eventual residual BPH tissue (**Fig. 4**) or fibrous formation (**Fig. 5**) occupying the prostatic fossa. 

**Fig. 6:**
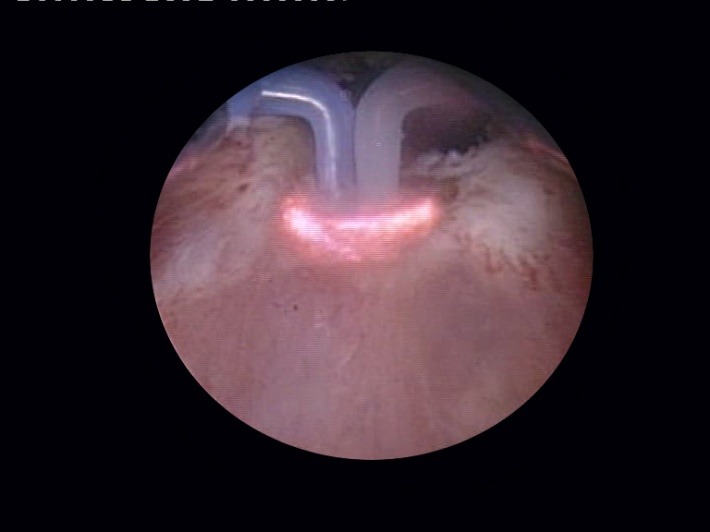
Obstructed bladder neck opening by plasma-button vaporization

The technique starts with the opening up of the obstructed bladder neck using the plasma-button vaporization (**Fig. 6**), either directly (if the communication with the bladder is well visualized) or by following the trajectory of the safety guidewire. 

**Fig. 7:**
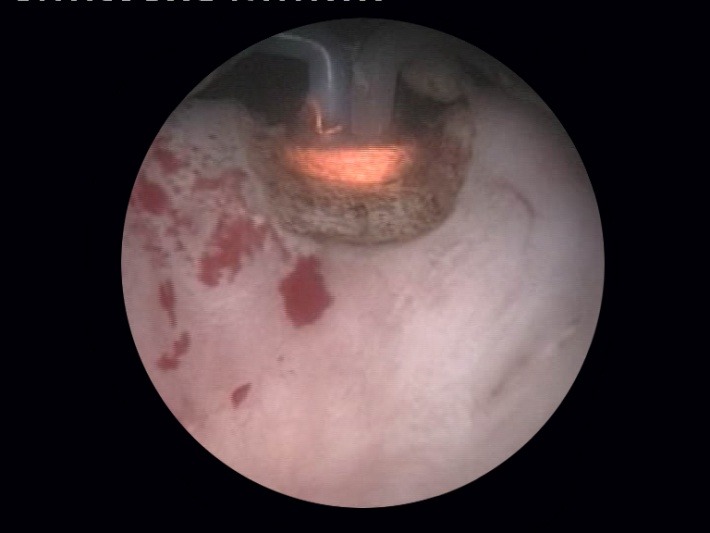
Bladder neck scar ablation at 6 o’clock

**Fig. 8:**
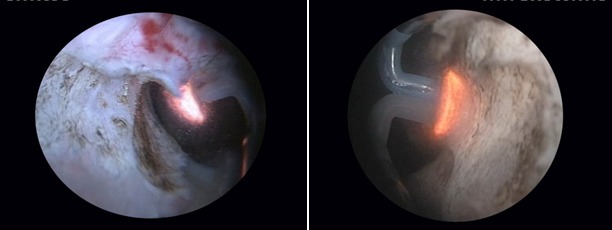
3 and 9 o’clock sclerotic tissue bipolar vaporization

**Fig. 9:**
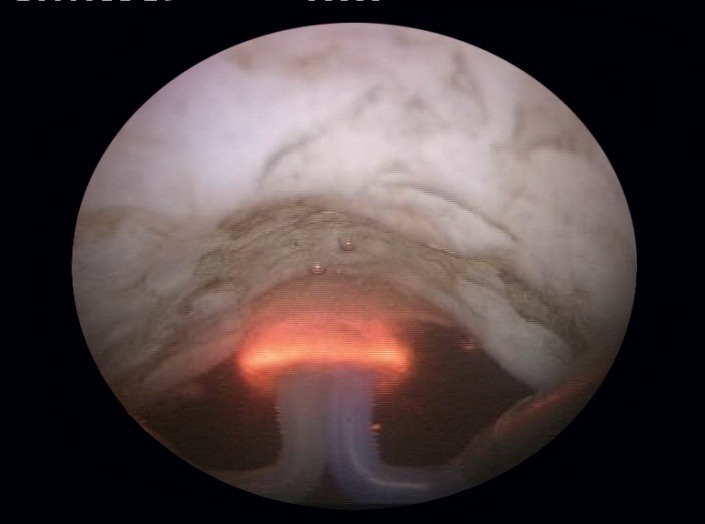
Fibrous tissue removal at the 12 o’clock position

 As soon as the resectoscope reaches the bladder, the bipolar vaporization process is continued in a circumferential manner, aiming to enlarge the bladder neck. Basically the same as during the simple resection, the scarring tissue is first ablated at the 6 o’clock position (**Fig. 7**), followed by 3 and 9 o’clock vaporization (**Fig. 8**) and finishing of at 12 o’clock (**Fig. 9**).

**Fig. 10:**
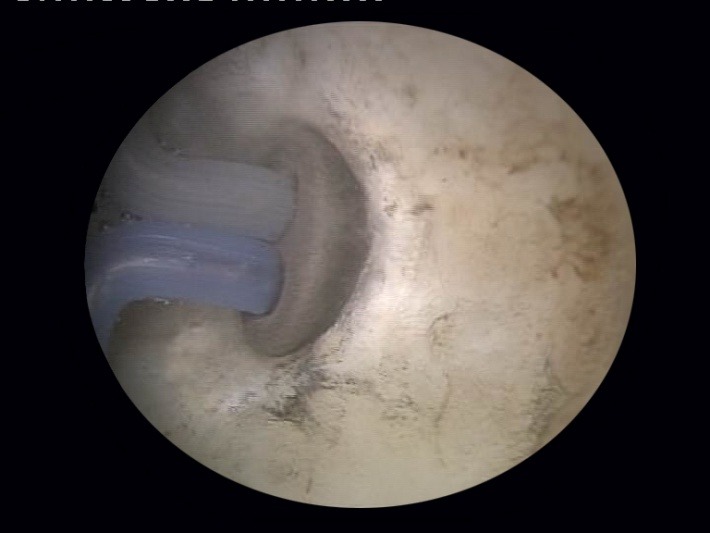
Bleeding sources’ coagulation at the end of surgery

Finally, although remotely necessary, the coagulation of any remaining hemorrhagic sources is completed at the end of the intervention (**Fig. 10**). Since the vaporization and coagulation phenomenona are virtually concomitant, thus leaving little need for additional hemostasis, this surgical stage is usually applied just as a precaution.

**Fig. 11:**
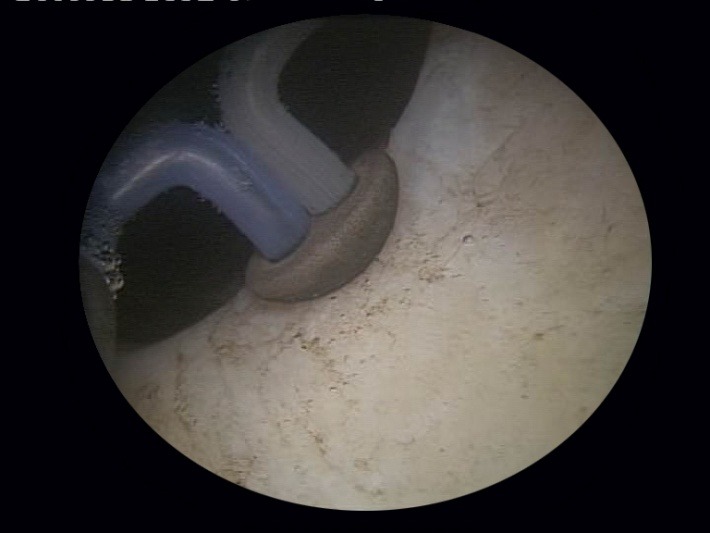
Clear fibers and smooth surface of the prostatic capsule

**Fig. 12:**
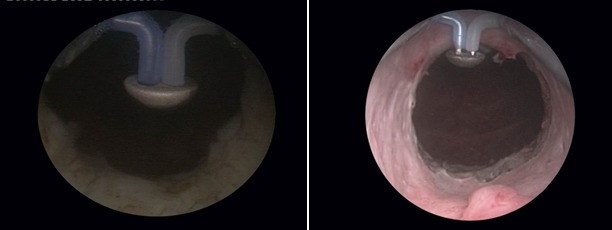
Wide prostatic fossa and bladder neck opening subsequent to the BPV approach

 Most importantly, the final endoscopic aspect obtained due to the BPV therapeutic alternative displays easily observable fibers and neat surface of the prostatic capsule (Fig. 11) as well as a wide prostatic fossa and bladder neck opening (fig. 12). Overall, the vaporization area is characterized by a complete lack of irregularities, bleeding sources, tissue debris or residual obstruction.

**Details of surgical technique – How to do it right**

Practically speaking, the fibrous tissue is gradually removed, beginning from the surface of the scar block and continuing until reaching down at the prostatic capsule. During the course of this so-called "hovering" technique, a strictly close contact must be maintained between the hemispheric shape electrode surrounded by a plasma corona and the sclerotic surface. Consequently, a constant vaporization effect is obtained, providing the conditions for a fast and efficient scar tissue ablation to be produced. 

While targeting to optimize the scar tissue ablation process, a relatively deep digging of the "button" electrode into the surface of the sclerotic block should be maintained throughout the entire ablative procedure. The movements of the working element should be carried out with a preferably low speed in order to ensure a more efficient vaporization effect, because during the BPV technique, slower actually means faster. It is also interesting to point out that the plasma vaporization approach provides constant contact tissue ablation during both active as well as passive movements of the bipolar resectoscope, thus substantially contributing to the effectiveness and overall speed of the intervention. 

The ablation is continued until reaching the prostatic capsule, a surgical stage that must be promptly identified in order to avoid significant intraoperative bleeding. It is also useful to observe any restant scar formation and completely remove it while intending to prevent future recurrences. A special attention should be particularly given to the bladder neck, which must display a wide opening at the end of the vaporization process. 

More over, the prostatic fossa should be carefully inspected and any residual BPH tissue must the thoroughly vaporized. The most relevant premises for a favorable postoperative clinical outcome are constituted by the obtaining of a large prostatic fossa and widened bladder neck, free from any type of irregularity or obstruction and displaying a clean prostatic capsule, from the veru montanum up to the bladder neck. 

**Technical settings and technological advances – What difference do they make?**

The standard equipment used for this procedure includes a bipolar energy source, the Visera video system, saline continuous flow irrigation, the OES-Pro resectoscope and the "button" shaped vapo-resection electrodes (Olympus Europe, Hamburg, Germany). From the technical settings point of view, the first generation energy source (UES-40 generator) usually uses 280 W for vaporization and 160 W for coagulation. Unfortunately, repeated pauses during the vaporization process are necessary every 8-12 seconds in order to avoid overheating the system, thus disrupting the procedure at possibly inappropriate moments, prolonging the operation time and seriously impeding on the fluency of the BPV technique [**14**]. 

More over, the sclerotic tissue encountered in BNS patients turns out to be rather resistant to the bipolar vaporization phenomenon and often requires a power increase of up to 300-320 W, thus raising certain concerns as to the surgical safety in general and tissue penetration depth in particular during the BPV procedure. 

The solution to this issue came with the introduction in clinical practice of a second generation bipolar device (ESG-400), which managed to provide the conditions for a continuous plasma-button vaporization to be achieved, unmarked by time limitations, device related drawbacks or ablation process impairments. From this perspective, the BPV specific operation length and overall fluency were substantially improved subsequent to this technological advancement [**15**]. 

Last but not least, the optimal power settings specific for this new generator were reduced to 200 W for vaporization and 120 W for coagulation, consequently outlining a superior plasma vaporization safety profile while maintaining or even raising the therapeutic efficacy. Also, no voltage increase was found as necessary when dealing with scar tissue formations, while, unlike with the previous energy source, the plasma ignition phenomenon truly occurred in an instantaneous manner. 

**Practical advantages – Why is BPV a real step forward**

One of the most important technical advantages of this type of approach is constituted by the remarkably clear visualization of the macroscopic characteristics of the specific tissue layers. The plasma-button vaporization procedure does not produce significant coagulation artifacts, nor does it leave behind bothering burning marks or surface irregularities. Subsequently, every tissue layer that is removed can be closely observed, thus ensuring a satisfactory safety of the ablation phenomenon. As a result, the sclerotic formation is gradually ablated until the specific visual features of the prostatic capsule are noticed (regularly disposed longitudinal white-pinkish fibers). 

The endoscopic vision throughout the intervention remains of good quality due to the concomitant vaporization and coagulation processes resulting in very few if any hemorrhagic sources. Also, the fact that the moment of surgery when reaching the prostatic capsule can be easily identified prevents capsular perforations as well as the opening of large vessels or venous sinuses, thus further reducing the bleeding risks associated with this therapeutic alternative. 

The overall operation time length is quite short, especially when applying the continuous plasma vaporization mode. There is hardly any intraoperative hemorrhage, no resection specimens to extract and the usually small quantity of fibrous tissue is quickly removed. Last but not least, the plasma vaporization is generally an easy-to-learn procedure, which largely benefits from the excellent endoscopic visibility related to the reduced bleeding as well as from the overall increased safety of this type of intervention. Subsequently, a rather short learning curve of about 10 procedures has been generally outlined, as the plasma-button vaporization technique was widely described by urologists as easier than the standard monopolar resection. 

**Plasma-button vaporization, a match for previously used therapeutic approaches?**

Certainly, a parallel to other treatment alternatives should be outlined before drawing some conclusions regarding the presently discussed BPV technique. For once, the transurethral incision by either cold-knife or laser (Holmium or Thulium) already begins with the substantial drawback of leaving behind the scar tissue formation in place. Practically, it is quite easy to imagine that, although properly incised, the sclerotic tissue block will regenerate, contract around the bladder neck and ultimately produce stenosis and subsequent lower urinary tract obstruction once again. 

Consequently, the clinical results specific for the cold-knife incision described in the literature data turned out to be rather unsatisfactory [**16**]. On the other hand, the Ho:YAG [**17**] laser incision has been occasionally reported as a somewhat promising therapeutic solution but eventually failed to gain acknowledgement as part of the standard treatment armamentarium in secondary BNS cases. The same as with all bladder neck incision techniques, the fact that, although incised, the fibrous structure blocking the bladder neck is left in place, represents the premises for future recurrences and a subsequently poor clinical outcome [**18**]. 

From another perspective, the standard monopolar transurethral resection (TUR), otherwise representing the conventional BPH therapy, has been also routinely applied in bladder neck contracture patients as well [**19**]. From the very start, this alternative is marked by a significant technical disadvantage: the regular loop resection is quite inappropriate for ablating small volume formations such as the fibrous block present in secondary BNS cases. Practically, the surface of the sclerotic diaphragm is rather close to the prostatic capsule, the loop resection depth is difficult to control in this situation and consequently, the capsular perforation and hemorrhagic risks are substantial. 

Additionally, although the premises for a complete scar tissue removal seem to be satisfied during conventional resection, the follow-up parameters and clinical evolution specific for this otherwise simple technique appear to display somewhat contradictory and basically unsatisfactory outcomes. In other words, while some authors emphasized a generally favorable result secondary to monopolar TUR [**20**], others underlined much less impressing results in terms of BNS recurrence, occasionally even inferior to the simple endoscopic incision [**21**]. 

Therefore, while keeping in mind the above mentioned partially failed attempts to provide a definite and successful treatment alternative in secondary bladder neck contracture patients, there seems to be significant room for improvement in the therapeutic management of this particular type of pathology and the BPV approach could be the answer that has been looked for during the past years. 

## Conclusions

The plasma-button vaporization appears to constitute a feasible solution in secondary BNS cases. The technical advantages displayed by this newly introduced procedure lay out the premises for a safe and complete sclerotic tissue ablation to be achieved. While obeying by certain useful "trips and tricks", the bipolar vaporization may be described as a simple, easy-to-learn and efficient modality of disposing of the obstructing scar formation occupying the bladder neck area. 

 The so far lack of evidence based successful BNS management based on retrograde incision or resection appears to have found a simple end efficient solution in the bipolar vaporization approach. The reduced learning curve, minimal bleeding risks, superior endoscopic vision, excellent ablation control and radical scar tissue removal appear to constitute rather convincing arguments in favor of this type of procedure. 

The recent technological advances in this field, together with a well established technique, further outline an optimistic perspective concerning this promising technique. Although further clinical research is definitely required to establish the long term clinical viability of this therapeutic modality, BPV seems to intuitively overcome its predecessors in terms of surgical safety, fibrous tissue radical ablation and postoperative evolution.

## References

[1] Moudouni  SM, Nouri  M (1999). Secondary sclerosis of the prostatic compartment after surgical treatment of benign prostatic hypertrophy. Ann Urol (Paris)..

[2] Sataa  S, Yassine  N (2009). Bladder neck sclerosis after surgical or transurethral resection of the prostate: a report of 40 cases. Tunis Med.

[3] Sevriukov  FA, Puchkin  AB (2007). Transurethral electrosurgery of a new generation (TURis) in the treatment of the lower urinary tract and prostate diseases. Urologia.

[4] Herrando  C, Batista  JE (1994). Bladder neck sclerosis after transurethral resection of the prostate. "Study Group of the Puigvert Foundation". Actas Urol Esp.

[5] Bach  T, Herrmann  TR (2007). Bladder neck incision using a 70 W 2 micron continuous wave laser (RevoLix). World J Urol.

[6] Al-Singary  W, Arya  M (2004). Bladder neck stenosis after transurethral resection of prostate: does size matter?. Urol Int.

[7] Salant  RL, Cohen  MS (1990). Neodymium:YAG laser treatment of postoperative bladder neck contractures. Urology.

[8] Martín-Laborda y Bergasa  F, Vallejo Herrador J (1995). Endoscopic cervicotomy: elective treatment in bladder neck sclerosis. Arch Esp Urol.

[9] Geavlete  B, Multescu  R (2010). Transurethral resection (TUR) in saline plasma vaporization of the prostate vs standard TUR of the prostate: 'the better choice' in benign prostatic hyperplasia?. BJU Int.

[10] Geavlete  B, Multescu  R (2011). Innovative technique in nonmuscle invasive bladder cancer-bipolar plasma vaporization. Urology.

[11] Geavlete  B, Multescu  R (2011). Bipolar plasma vaporization of secondary bladder neck sclerosis. 31st Congress of the Societe Internationale d’Urologie, 16-20 October 2011, Berlin. Urology.

[12] Geavlete  B, Stănescu  F (2012). Bipolar plasma vaporization in secondary bladder neck sclerosis—initial experience with a new technique. J Med Life.

[13] Geavlete  B, Moldoveanu  C (2013). Bipolar plasma vaporization versus standard transurethral resection in secondary bladder neck sclerosis: a prospective, medium-term, randomized comparison. Ther Adv Urol.

[14] Geavlete  B (2012). Continuous plasma vaporisation. A new step forward in BPH endoscopic treatment. European Urology Today.

[15] Geavlete  B, Stanescu  F (2013). Continuous versus conventional bipolar plasma vaporization of the prostate and standard monopolar resection – A prospective, randomized comparison of a new technological advancement. BJU Int.

[16] Pérez Arbej  JA, Cameo Rico  MI (1991). Postoperative sclerosis of the bladder neck: surgical treatment. Arch Esp Urol.

[17] Bader  MJ, Tilki  D (2010). Ho:YAG-laser: treatment of vesicourethral strictures after radical prostatectomy. World J Urol.

[18] Bader  MJ, Tilki  D (2010). Ho:YAG-laser: treatment of vesicourethral strictures after radical prostatectomy. World J Urol.

[19] Popken  G, Sommerkamp  H (1998). Anastomotic stricture after radical prostatectomy. Incidence, findings and treatment. Eur Urol.

[20] Jocius  KK, Sukys  D (2002). Treatment of bladder neck obstruction (sclerosis): personal experience and literature review. Medicina (Kaunas).

[21] Sikafi  Z, Butler  MR (1985). Bladder neck contracture following prostatectomy. Br J Urol.

